# Integrative Analysis of Somatic Mutations Altering MicroRNA Targeting in Cancer Genomes

**DOI:** 10.1371/journal.pone.0047137

**Published:** 2012-10-16

**Authors:** Jesse D. Ziebarth, Anindya Bhattacharya, Yan Cui

**Affiliations:** 1 Department of Microbiology, Immunology and Biochemistry, University of Tennessee Health Science Center, Memphis, Tennessee, United States of America; 2 Center for Integrative and Translational Genomics, University of Tennessee Health Science Center, Memphis, Tennessee, United States of America; H. Lee Moffitt Cancer Center & Research Institute, United States of America

## Abstract

Determining the functional impact of somatic mutations is crucial to understanding tumorigenesis and metastasis. Recent sequences of several cancers have provided comprehensive lists of somatic mutations across entire genomes, enabling investigation of the functional impact of somatic mutations in non-coding regions. Here, we study somatic mutations in 3′UTRs of genes that have been identified in four cancers and computationally predict how they may alter miRNA targeting, potentially resulting in dysregulation of the expression of the genes harboring these mutations. We find that somatic mutations create or disrupt putative miRNA target sites in the 3′UTRs of many genes, including several genes, such as *MITF*, *EPHA3*, *TAL1*, *SCG3*, and *GSDMA*, which have been previously associated with cancer. We also integrate the somatic mutations with germline mutations and results of association studies. Specifically, we identify putative miRNA target sites in the 3′UTRs of *BMPR1B*, *KLK3*, and *SPRY4* that are disrupted by both somatic and germline mutations and, also, are in linkage disequilibrium blocks with high scoring markers from cancer association studies. The somatic mutation in *BMPR1B* is located in a target site of miR-125b; germline mutations in this target site have previously been both shown to disrupt regulation of *BMPR1B* by miR-125b and linked with cancer.

## Introduction

The genomes of most adult human cancers contain thousands of somatic mutations [1], and a critical aspect of cancer research is determining which of these somatic mutations have crucial functional impact on biological processes related to tumorigenesis and metastasis [2], [3], [4]. Until recently, efforts to sequence cancer genomes have focused on the impact of mutations in coding regions and identifying non-synonymous point mutations, small frameshift deletions, or large genomic rearrangements that may, for example, create fusions genes [5], [6]. With the rapid advances in sequencing technologies, it has become possible to sequence and compare whole genomes of normal and cancer tissues from the same individual to identify somatic mutations [7]. Recently, the entire genomes of normal and cancer tissues in patients with lung cancer [8], melanoma [9], small cell lung cancer (SCLC) [10], and prostate cancer [11] have been sequenced, providing somatic mutations in these cancers in both coding and non-coding regions. However, there has, to this point, been limited investigation of the effect of non-coding somatic mutations on cancer pathogenesis.

One effect of somatic mutations in non-coding regions that has the potential to significantly impact cellular functions associated with cancer is the alteration of microRNA (miRNA) targeting. MicroRNAs are small, non-coding RNAs that function as posttranscriptional regulators of mRNA expression, typically by inhibiting translation or causing the degradation of their mRNA targets. Many miRNAs are up- or down-regulated in cancers, indicating that they act as oncogenes or tumor suppressors, respectively; and miRNA expression profiles have been used to accurately classify cancer subtypes [12]. MicroRNAs have been shown to control many important cellular processes that are altered in cancers, including differentiation, proliferation, and apoptosis [13]. The function of miRNAs is particularly sensitive to genetic variants because complementarity between the seed region of the miRNA and an mRNA sequence is often required for miRNA targeting [14]. Therefore, it is not surprising that germline mutations that disrupt miRNA targeting have been found to play important roles in many diseases [15], [16], [17], [18] including several types of cancer [19], such as melanoma [20], leukemia [21], [22], and breast cancer [23], [24], as well as in oncogenic transformation [25]. Germline mutations that alter miRNA target sites have also been investigated as being the functional causative variants that underlie the results of genome-wide association studies (GWAS) [26], [27]. Recently, a somatic mutation in the 3′UTR of *TNFAIP2*, a known target of the *PRAM1* oncogene, creates a new miRNA target site that results in a reduction of *TNFAIP2* expression in a patient with acute myeloid leukemia [28]. This example illustrates the potential for somatic mutations to alter miRNA targeting and contribute to pathogenesis, but there has, to this point, been limited investigation of somatic mutations located in miRNA target sites.

**Figure 1:**
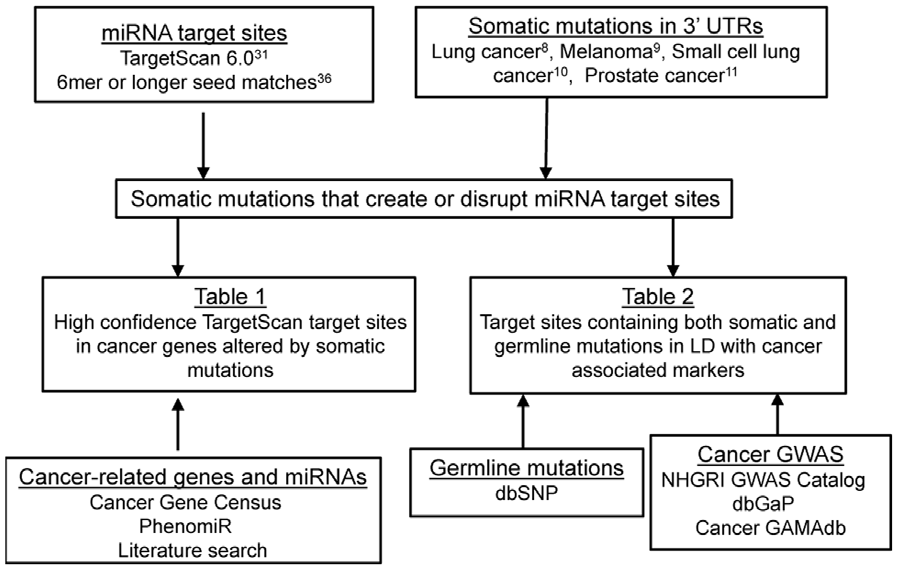
Overview of the study. Somatic mutations within putative miRNA target sites are linked with cancer-related genes and miRNAs as well as the results of cancer association studies.

Here, we systematically examine how somatic mutations may alter miRNA targeting (Figure 1). First, we collect somatic mutations in 3′UTRs, the genomic regions that are typically considered to be the most common binding sites of miRNAs, obtained from whole genome sequences of four cancers and analyze the patterns of these 3′UTR mutations. Next, we computationally predict how 3′UTR somatic mutations alter miRNA target sites and identify which of these somatic mutations may be particularly relevant to cancer pathogenesis. We determine somatic mutations that are both located within genes that have been linked with cancer and alter putative target sites of cancer-related miRNAs. We also attempt to link alteration of miRNA targeting with cancer through integration of these somatic mutation with the results of association studies. We identify three miRNA target sites that are altered by both somatic and germline mutations in linkage disequilibrium blocks with high scoring markers identified in GWAS of cancers.

## Results

### Patterns of somatic mutations in 3′UTRs

**Figure 2:**
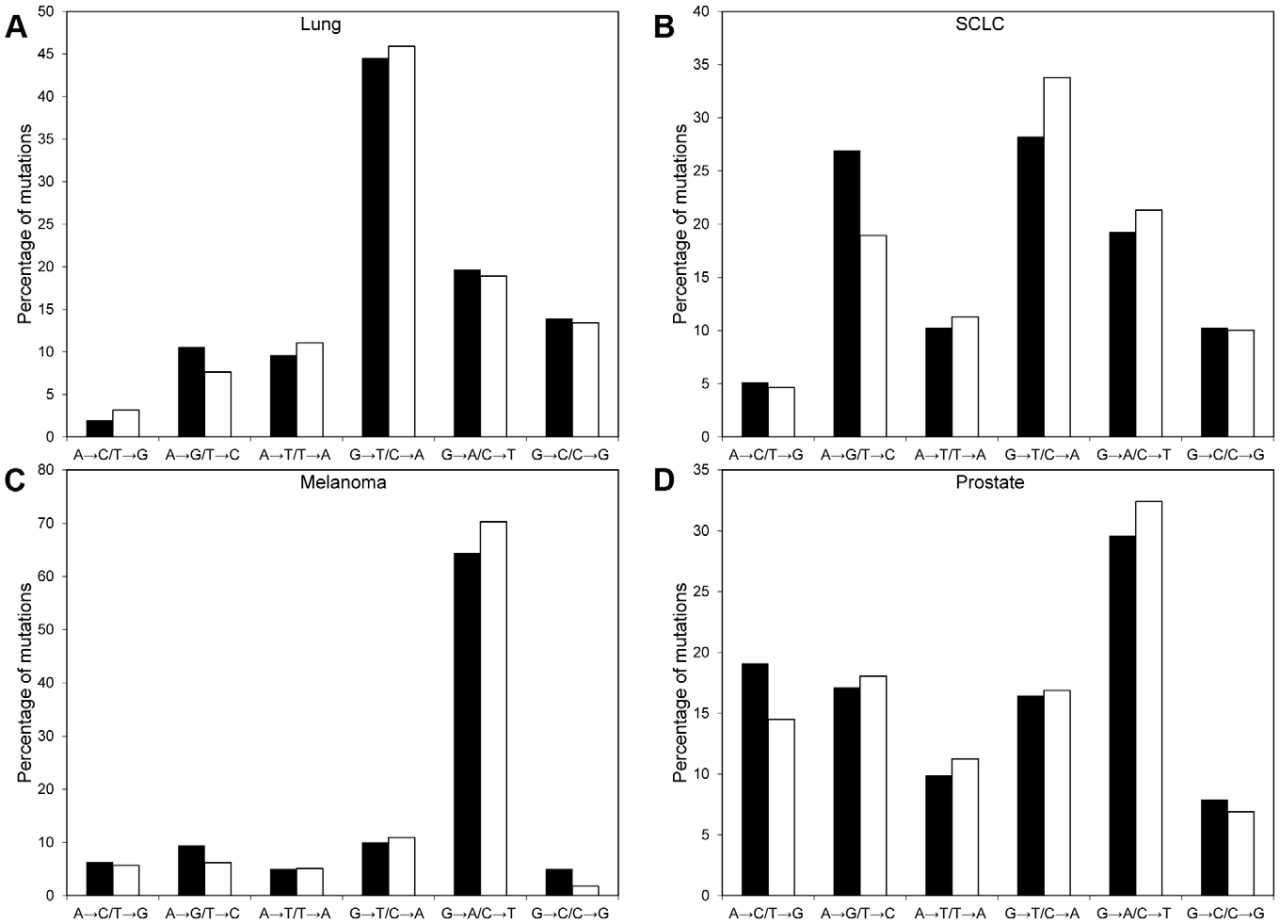
Frequency of single base substitutions. The percentage of each class of substitution among somatic mutations in 3′UTRs (black bar) or across the entire genome (white bar) is shown for (A) lung cancer, (B) SCLC, (C) melanoma, and (D) prostate cancer.

We collected a total of 610 somatic mutations in 3′UTRs from four cancers (SCLC, melanoma, lung, and prostate). Excepting prostate cancer, somatic mutations were determined from whole genome sequencing of single samples; seven samples were sequenced for prostate cancer. None of the somatic mutations in 3′UTRs were identified in multiple cancer types. Only 1 (a T>C substitution at 30693148 in the 3′UTR of *TUBB* that was found in two prostate cancer samples) of the 152 (0.66%) somatic mutations in 3′UTRs identified in prostate cancer was found in multiple samples. The occurrence of somatic mutations in multiple prostate cancer samples across the entire genome was similarly rare, as only 116 of the 28626 (0.41%) of the somatic mutations in prostate cancer were found in multiple samples genome-wide. To compare the types of substitutions that occurred in each cancer type, we calculated the frequency of each class of single base substitution (Figure 2). The distributions of substitutions in 3′UTRs varied across types of cancers. For example, the majority of melanoma substitutions were G>A/C>T, while the most prevalent mutations in both lung and SCLC samples were G>T/C>A substitutions. These trends agreed with the rates of the mutations found across all regions of the genome for each type of cancer, and, in general, the percentage of mutations for each type of substitution were similar for 3′UTRs and for the entire genome. Together, these results indicate that mutations in 3′UTRs have similar causes (e.g., ultraviolet exposure for melanoma, smoking for lung cancer) as the mutations in the entire genome.

**Figure 3:**
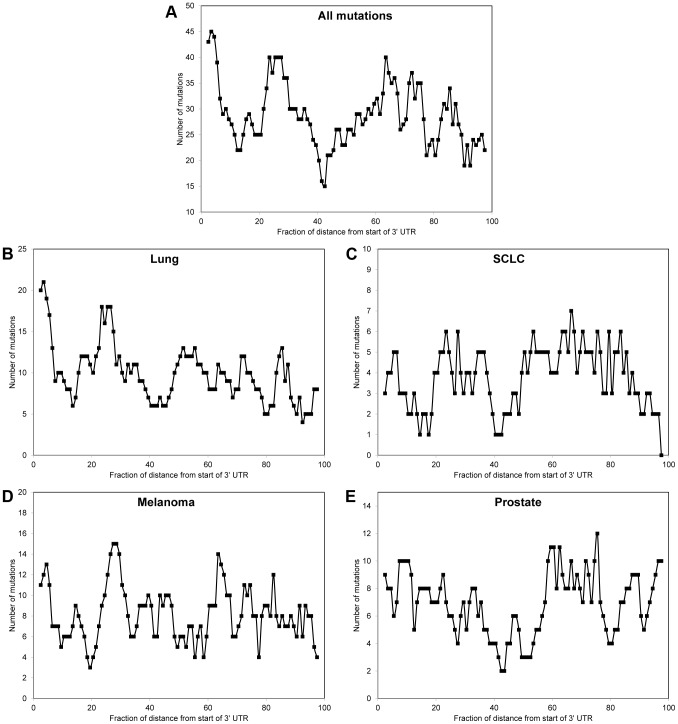
Location of somatic mutations in 3′UTRs. For each somatic mutation, the percentage of the distance from the start of the 3′UTR to the somatic mutation compared to the total length of the 3′UTR was calculated. The figure shows the number of mutations in rolling windows of 5% of the 3′UTR length for somatic mutations in (A) all cancer types, (B) lung cancer, (C) SCLC, (D) melanoma, and (E) prostate cancer.

We also investigated if somatic mutations in 3′UTRs were more likely to be located at the 5′ end or 3′ end of the 3′UTR. For each somatic mutation, we compared the distance from the start of the 3′UTR (i.e, the end of the final exon) to the mutation to the total length of the 3′UTR. We then counted the number of somatic mutations in different sections of the 3′UTRs using a rolling window with a width of 5% and found that the number of somatic mutations varied considerably along the 3′UTR (Figure 3). The overall pattern of the distribution of all of the somatic mutations (Figure 3a) most closely matches that obtained from lung cancer (Figure 3b), the study that produced the largest number of mutations. In lung cancer (Figure 3b), there are many mutations immediately downstream of the end of the final coding exon, with the number of mutations sharply decreasing as the distance approached 10% of the 3′UTR length.

### Somatic mutations in 3′UTRs alter miRNA targeting

While a complete understanding of how the mRNA targets of a miRNA are selected has yet to be elucidated, sequence complementarity between nucleotides at the 5′ end, or seed region, of the mature miRNA sequence and a mRNA target site, which is typically in the 3′UTR, is common to many miRNA-mRNA pairs. Dozens of computational methods for predicting the targets of miRNAs have been developed, based on complementarity, as well as other criteria including conservation of the target site across species, target site accessibility in the secondary structure of the mRNA, the sequence context of the target site, and the thermodynamics of binding [29], [30]. We used two methods to identify somatic mutations with the potential to impact miRNA targeting (Table S1). First, we calculated context+ scores using the latest version of TargetScan [31], one of the most widely used and highest performing miRNA prediction tools [32], [33], for two sets of 3′UTR sequences, one containing the allele found in the normal tissue and one containing the allele found in cancer tissue. We then identified somatic mutations that were located within target sites predicted by TargetScan and impacted context+ scores. Second, we attempted to create a more inclusive list of 3′UTR somatic mutations that impact miRNA targeting by determining the mutations that alter 6mer, 7mer, or 8mer sites complementary to miRNA seeds. This second approach was motivated by recent analysis of mRNA sequences targeted by miRNAs in CLIP-Seq experiments in human [34] and HITS-CLIP experiments in mouse [35] that found that while longer (e.g., 7 nt and 8 nt) matches between the mRNA sequence and miRNA seed had higher specificities, the majority of functional target sites contained only 6 nt matches [36].

Given the large number of unique miRNA seeds, we expected to find that most somatic mutations either disrupted or created at least a 6mer match to a miRNA seed (Table S1). 608 of the 610 somatic mutations in 3′UTRs altered at least a 6mer long potential miRNA binding site and 525 mutations altered context+ scores calculated by TargetScan 6.0 for at least one miRNA. We then attempted to identify somatic mutations that had a high priority of having a role in cancer pathogenesis. First, we selected only miRNA-mRNA pairs for which the somatic mutation resulted in a magnitude change greater than 0.2 for the context+ score of a miRNA targeting the mRNA, providing the somatic mutations in target sites that were in the top 15% of those most likely to be functional based on the context+ score. Next, we limited the impacted putative target sites based on the miRNA and removed miRNAs that either had low expression (fewer than 100 total reads) in the RNA-Seq experiments collected in miRBase [37] or have not been previously associated with cancer in the PhenomiR database [38]. Finally, we used the Cancer Gene Census [39] and other literature sources to identify genes that are known tumor suppressors, oncogenes, or have other functional associations with cancer. Table 1 contains a selection of the somatic mutations that altered miRNA targeting and met these criteria. We also examined tissue- and cancer-specific miRNA expression to identify miRNAs that have been shown to be highly expressed in the particular tissue or cancer in which the somatic mutations were identified (Table S1). Several of the somatic mutations in Table 1, including those in *TAL1*, *BMPR1B*, *KDM5A*, *SCG3*, and *BCAS3* impacted target sites of miRNAs that have been shown to be expressed in the same tissue in which the miRNA was identified.

**Table 1:** Selected somatic mutations that alter miRNA target sites in cancer-related genes.

Somatic mutation^a^	Cancer type	Gene	Association with cancer	miRNA	Effect of mutation
**Chr1:g.47684484CC>GT**	**Melanoma**	***TAL1***	**Oncogene associated with poor prognosis in T-cell acute lymphoblastic leukemia ** [**40**]	**miR-185-3p ** [**78**]	**Disrupts target site**
**Chr3:g.70015386T>C**	**Prostate**	***MITF***	**Aberrant regulation of ** ***MITF*** ** occurs in melanoma (18316599 ** [**45**]	**miR-18a-5p ** [**79**]	**Creates target site**
**Chr3:g.89448841C>A**	**Prostate**	***EPHA3***	**Under-expressed in prostate cancer cell lines ** [**44**]	**miR-539-3p ** [**80**] **, miR-485-3p ** [**81**]	**Modifies target site (m6b→m7b)**
**Chr4:g.96075969G>T**	**Lung**	***BMPR1B***	**Variants in miR-125b binding site associated with breast and prostate cancer ** [**46]**, [82]	**miR-125a-5p ** [**79**]	**Disrupts target site**
Chr9:g.23692113A>G	SCLC	*ELAVL2*	Overexpressed in small-cell lung cancer [83]	miR-532-3p [84]	Creates target site
Chr12:g.389785A>T	Melanoma	*KDM5A*	Up-regulated in gastric cancer and required for sustained proliferation of cancer cells [85]	miR-505-5p [81]	Creates target site
Chr12:g.53604521C>A	Melanoma	*RARG*	Decreased expression in melanoma [86]	miR-766-5p [87]	Modifies target site (m7b→m6b)
**Chr15:g.52011992G>A**	**Melanoma**	***SCG3***	**Overexpressed in SCLC ** [**41**]	**miR-330-3p ** [**88**]	**Modifies target site (m7b→m6b)**
**Chr17:g.38133463C>A**	**Lung**	***GSDMA***	**Potential tumor suppressor that is overexpressed in gastric cancer ** [**42**]	**miR-92a-1-5p ** [**79**]	**Disrupts target site**
Chr17:g.59469532A>G	Melanoma	*BCAS3*	Over-expressed in brain tumors (18030336 [89]) and breast cancer [90]	miR-361-3p [88]	Creates target site
Chr19:g.31767186G>A	Lung	*TSHZ3*	Potential tumor suppressor under-expressed in breast and prostate cancer [91]	miR-30b-3p [79]	Modifies target site (m6c→m7b)

a :Mutations in bold type indicate somatic mutations in genes over-expressed in cancers that create or enhance miRNA target sites or somatic mutations in genes under-expressed in cancer that disrupt or hinder miRNA target sites.

Of particular interest are oncogenes with somatic mutations that disrupt miRNA targeting and tumor suppressors with somatic mutations that create new miRNA targets, as these mutations could potentially explain the respective up- and down-regulation of these genes in cancers (Mutations meeting this criterion are shown in bold in Table 1). For example, increased expression of *TAL1* [40], *SCG3* [41] and *GSDMA* [42], [43] has been observed in cancers, and somatic mutations in the 3′UTRs of these genes disrupt putative targets of miRNAs that have been associated with cancer. The disruption of these target sites may prevent regulation of the levels of these genes by miRNAs, leading to higher expression. In contrast, *EPHA3* [44] and *MITF* [45] are under-expressed in cancers or have been shown to act as tumor suppressors; the somatic mutations may create new target sites that lead to increased inhibition of translation or degradation of the mRNAs. Notably, one of the somatic mutations selected by this method impacted an experimentally validated target site of miR-125b in *BMPR1B* [46], which will be examined in more detail in the next section.

### GWAS- and CGAS-informed functional analysis of somatic mutations that alter miRNA targeting

**Figure 4:**
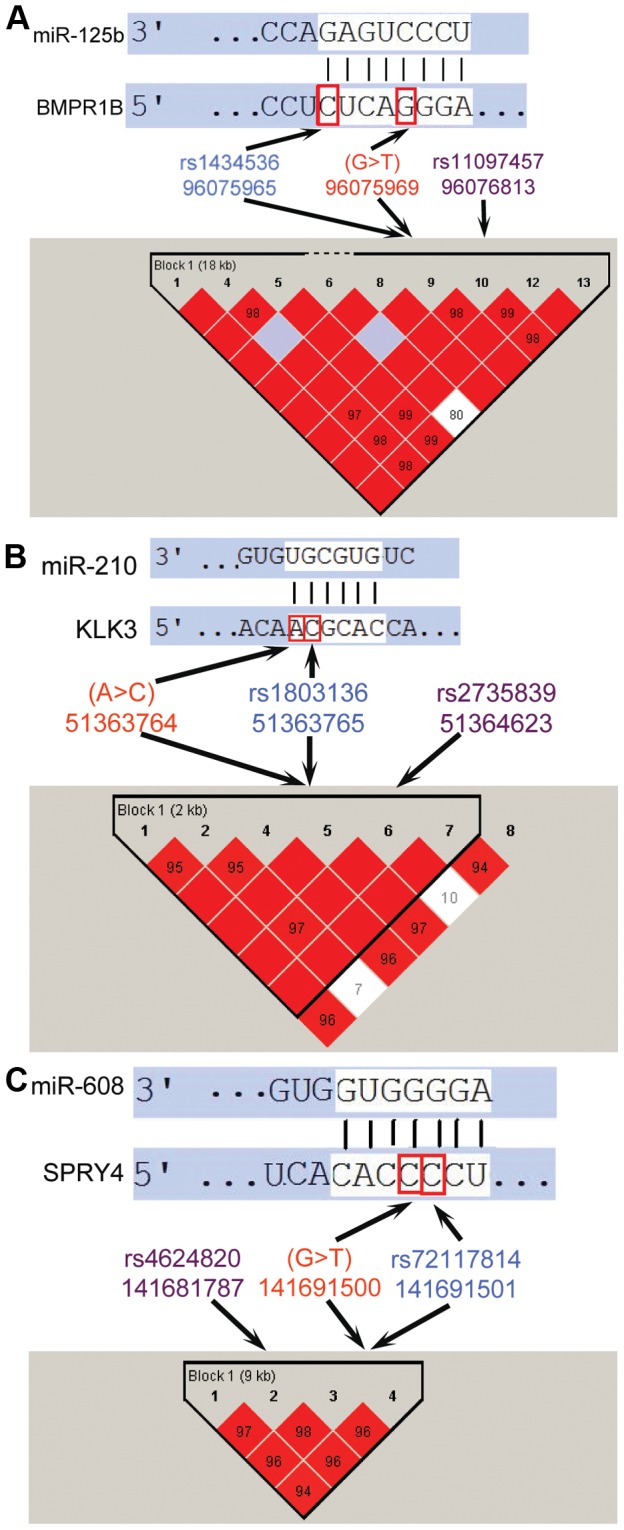
Disruption of miRNA target sites that are in linkage disequilibrium with high scoring markers (purple) from cancer association studies by both germline (blue) and somatic (red) mutations. (A) Disruption of a target site of miR-125b in the 3′UTR of *BMPR1B*. (B) Disruption of a target site of miR-210 in the 3′UTR of *KLK3*. (C) Disruption of a target site of miR-608 in the 3′UTR of *SPRY4*.

Genome-wide and candidate gene association studies have identified a large, and growing, number of genomic locations harboring germline mutations associated with increased risk for cancer. In many cases, the specific germline mutations that underlie these associations and their functional impact remain unknown; however, germline mutations that alter miRNA targeting have been identified as promising candidates for potentially explain the increased risk for several of cancers [19]. Therefore, we attempted to integrate the somatic mutations that alter miRNA targeting with germline mutations and the results of association studies. We sought to identify miRNA target sites in linkage disequilibrium with high scoring markers from association studies that are altered by both germline mutations and somatic mutations identified in cancers. Specifically, we identified both experimentally supported and computationally predicted miRNA target sites altered by somatic mutations that were also altered by germline mutations, and then, determined if the target was in the same haplotype block as high scoring markers from cancer association studies. Three genes, *BMPR1B*, *KLK3*, and *SPRY4*, contained miRNA target sites altered by both somatic and germline mutations that were in linkage disequilbrium blocks containing high scoring association study markers (Table 2 and Figure 4).

**Table 2:** Somatic mutations that alter miRNA targeting in linkage disequilibrium blocks of association study markers.

Somatic mutation	Cancer type	Gene	miRNA	Germline mutation in target site^a^	Association study marker^a^	LD block boundaries	Cancer type in association study	P-Value
Chr4:g.96075969G>T	Lung	BMPR1B	miR-125b	rs1434536 (4)	rs11097457 (844)	96061509–96091524	Breast Neoplasms [46]	1.97E-04
Chr19:g.51363764A>C	Prostate	KLK3	miR-138, miR-210, hsa-miR-675-5p	rs1803136 (1)	rs2735839 (859)	51361757–5134623	Prostatic Neoplasms [49]	2.00E-18
Chr5:g.141691500G>T	Prostate	SPRY4	miR-608	rs72117814 (1)	rs4624820 (9712)	141664185–141693593	Testicular Neoplasms [53]	3.00E-13

a : The number in parentheses below the name of the polymorphism is the distance, in bp, from the germline mutation to the somatic mutation.

The 3′UTR of *BMPR1B* contains a binding site for miR-125b that is disrupted by both a somatic mutation that was identified in lung cancer (chr4:g.96075969G>T) and a germline SNP (rs1434536). This target site is also in a haplotype block with rs11097457, one of the top 100 highest scoring markers in the Cancer Genetic Markers of Susceptibility (CGEMS) study, which is associated with breast cancer risk [46] (Figure 4a). The R^2^ value for correlation between rs11097457 and rs1434536 in the 1000 Genomes Project [47] is 0.82. The targeting of *BMPR1B* by miR-125b and the possibility that genetic variants disrupt this target site and play a role in cancer have been previously studied [46]. Saetrom et al. found that rs1434536 was in strong linkage disequilibrium with two high scoring markers in a breast cancer association study, confirmed the association in an independent breast cancer cohort, and showed that the SNP disrupted regulation of *BMPR1B* by miR-125b.

Both a somatic mutation (chr19:g.51363764A>C) and a germline mutation (rs1803136) in the 3′UTR of *KLK3*, a gene whose expression is commonly used as a diagnostic marker in prostate cancer [48], disrupted predicted target sites for miR-675, miR-138, and miR-210. These target sites were in the same linkage disequilibrium block, and only ∼850 basepairs away, from rs2735839 (Figure 4b), which was strongly associated with increased risk in a GWAS of prostate cancer [49]. Moreover, the somatic mutation (chr19:g.51363764A>C) was also identified in a patient with prostate cancer [11]. There has also been previous evidence that miR-675 [50], miR-210 [51] and miR-138 [52] regulate cancer cell proliferation. We also found a somatic mutation (chr5:g. 141691500G>T) and a germline mutation rs72117814 within a predicted binding site for miR-608 in the 3′UTR of *SPRY4* which was located in the same linkage disequilibrium block as rs4624820, a high-ranking marker in a testicular cancer GWAS [53], [54] (Figure 4c). *SPRY4* inhibits the mitogen-activated protein kinase pathway (MAPK) which is activated by the KITLG-KIT pathway, which has been associated with testicular cancer [53]. Because the germline mutations that disrupt target sites in *SPRY4* and *KLK3* are not included in the 1000 Genomes Project or HapMap data, we were not able to calculate the correlation between the germline SNPs and the high-ranking GWAS markers.

## Discussion

Recent sequencing of the entire genomes of normal and cancer tissues from the same individual have provided comprehensive lists of somatic mutations. While there have been several efforts to identify the functional impact of somatic mutations in coding regions [5], [55], non-coding somatic mutations have received relatively little attention, despite the importance of these regions to gene regulation. One report investigated the rates of non-coding somatic mutations in multiple myeloma and observed that many non-coding mutations were near coding regions with known somatic hypermutation and that the mutation frequency in some-non-coding regions was greater than that expected by chance [56], but the functional impact of these non-coding mutations was not investigated. Here, we made an initial effort to identify non-coding somatic mutations that have the potential to cause dysregulation of gene expression and contribute to cancer pathogenesis. Specifically, we focused on somatic mutations located in 3′UTRs and investigated how these mutations may alter miRNA targeting. We found that the distributions of the different types of single base substitutions among somatic mutations in 3′UTRs varied for different types of cancers, but agreed with the distributions across the entire genome in each cancer type (Figure 2). We also investigated the distribution of miRNAs across the 3′UTRs and found that, for lung cancer, there was a large number of somatic mutation located in the 3′UTR very near the final coding exon. The distribution of mutations across genes has been used to determine the selective application of DNA repair, and it has been shown that DNA repair is more common among transcribed strands compared to non-transcribed strands and to the 5′ end of genes compared with the 3′ end [9]. While the large number of somatic mutations in the 3′UTR near the final coding exon in lung cancer is only an initial result based on a relatively small number of somatic mutations, observation of similar behavior as more somatic mutations are identified may enable increased understanding of DNA repair in the 3′UTR.

One way in which somatic mutations within 3′UTRs may have a functional impact is if they impact miRNA targeting by disrupting or creating miRNA target sites. We specifically identified somatic mutations that are predicted to disrupt miRNA target sites within genes, including *TAL1*, *SCG3*, and *GSDMA*, that are over-expressed in cancer and mutations that are predicted to create new miRNA target sites within genes, including *MITF* and *EPHA3*, that are underexpressed in cancer. While it is straightforward to identify how somatic mutations may impact miRNA function through these two modes (oncogenes with disrupted sites and tumor suppressors with created sites), it is likely that dysregulation of miRNA function in cancer occurs through more complex relationships that may not be consistent for all types of cancer. For example, several miRNAs, including the miR-17-19b cluster [12], [57], [58], and genes, including *CDH1* [59], have been shown to have oncogenic properties in some cancer types while acting as tumor suppressors in others. Additionally, miRNAs increase the expression of their targets in some cases [60].

Greenberg et al. [61] investigated the global impact of somatic mutations in melanoma, lung cancer, and leukemia. They found that the mutations in melanoma decreased the binding of miRNAs to 3′UTRs, but did not observe as significant of a decrease in binding for somatic mutations in the other cancers. They attributed this result to UV-induced mutations found in melanoma being primarily Strong-to-Weak mutations (i.e., those mutations which reduce thermodynamic hybridization stability). While we focused on how the somatic mutations impacted complementarity between miRNA seeds and target sites, and not the impact of the mutations on binding energy, several of our results agreed with the conclusions by Greenberg et al. We found that the frequencies of the single base substitutions varied across cancer types (Figure 2), resulting in more Strong-to-Weak mutations in melanoma than other cancers. We can also use our results (Table S1) to compare with Greenberg et al. by calculating the ratio of the number of putative miRNA target sites disrupted by somatic mutations to the number of putative miRNA target sites created by the somatic mutations. The disrupted to created target site ratio is 1.18 for melanoma mutations, which is similar to the ratio found in SCLC (1.19) and higher than that found in prostate (1.12) and lung cancer (1.08), suggesting that it is possible that the somatic mutations in melanoma result in an overall decrease in miRNA binding in comparison with normal tissues and other cancers.

We attempted to identify important functional somatic mutations by leveraging the results of association studies. We identified target sites that contain both somatic and germline mutations and are in linkage disequilibrium blocks with high scoring markers from association studies of cancers. This procedure integrates two sources of information indicating the possibility that alteration of the target site plays a role in cancer; the germline mutation in the target site is a potential cause of the increased risk associated with the linked marker in the association study, while the somatic mutation in the target may play a role in tumorigenesis in other individuals. We identified three target sites located in *BMPR1B*, *KLK3*, and *SRPY4* that contain both somatic and germline mutations and are linked with association studies. Both the genes containing these somatic mutations and the miRNAs that target these sites have been previously associated with cancer. A 3′UTR somatic mutation in *BMPR1B* identified in a lung cancer patient disrupts the specific target site of miR-125b that has previously been investigated for its role in cancer [46]. The target site contains a SNP, rs1434536, that is in linkage disequilibrium with two high scoring markers in a breast cancer association study and results in disruption of the regulation of *BMPR1B* by miR-125b. The somatic mutation indicates a second path through which the regulation of the gene by miRNAs could be disrupted, potentially contributing to tumorigenesis. While there has not been such strong experimental support for mutations disrupting the regulation of *KLK3* [49]and *SPRY4* [53], [54] by miRNAs in cancer, both of these genes have strong associations with cancer. Levels of *KLK3* are commonly used for diagnosing prostate cancer [48], and the somatic mutation altering miRNA targeting of *KLK3* was identified in prostate cancer. *SPRY4* is involved in the KITLG-KIT pathway, which has been associated with cancer [53]. Additionally, two somatic mutations (chr12:g.88889449G>A and chr12:g.88887136G>A), in putative binding sites for miR-203 and miR-183, respectively, were located in the 3′UTR of *KITLG*. Expression of miR-183 has been shown to be correlated with expression of miR-203 [62], and both miRNAs are involved in suppression of expression of stem cell factors in cancer cells [62] and in proliferation of cancer [62], [63]. The *KITLG* somatic mutations are in a linkage disequilibrium block with rs995030, a marker SNP rs995030 which is strongly associated with testicular cancer risk [53]. Therefore, these somatic mutations in the 3′UTRs of *SPRY4* and *KITLG* are promising candidates for contributions to tumorigenesis by the dysregulation of the KITLG-KIT pathway.

While the current study was able to identify somatic mutations that may impact miRNA targeting and play a role in cancer pathogenesis, it is limited by several factors. First, all but one of the somatic mutations studied here was identified in a single patient, and, therefore, the mutations may not commonly be found in other patients or may not be generalizable to other populations and cancer etiologies. Second, due to the relatively small number of experimentally known miRNA binding sites and a lack of understanding of the specifics of miRNA targeting, this study was, in most cases, only able to identify somatic mutations that alter predicted miRNA target sites. Specifically, we focused on how somatic mutations impact sequences within 3′UTRs complementary to miRNA seeds, as these features have been the focus of most miRNA targeting prediction algorithms; however, this approach neglects how somatic mutations within other locations in a target site, such as 3′ compensatory sites, may impact binding. Additionally, while 3′UTRs have traditionally been believed to harbor the majority of miRNA target sites, several recent experiments have shown that 5′UTRs [64] and coding regions [65] also contain functional miRNA targets. In the coming years, we expect that improvements in sequencing technologies may be able to address these limitations, increasing understanding of how alteration of miRNA targeting by germline and somatic mutations plays a role in cancer and other diseases in the coming years. New experimental techniques, such as CLIP-Seq [34], [35], have the promise to provide both extensive lists of experimentally supported miRNA target sites and the basis for a more complete understanding of miRNA targeting, potentially improving computational target predictions. Also, the number of somatic mutations and cancer-associated markers from GWAS will likely continue to grow rapidly, and methods that integrate these resources will therefore become increasingly fruitful. In particular, increasing the number of known somatic mutations will allow for the identification of mutations that commonly occur in cancer. While we were to determine one target site (the target site of miR-125b in *BMPR1B*) that offered the combination of experimental support, disruption by both germline and somatic mutations, and links with association studies, these developing resources may soon enable the identification of many similar high priority miRNA targets.

## Materials and Methods

### Sources of somatic mutations in 3′UTRs

Somatic mutations were compiled from the supplementary material of the original papers for lung [8] and prostate [11] cancer and from the non-coding variants of the COSMIC database [66] for SCLC [10] and melanoma [9]. Somatic mutations were determined using SOLiD, for SCLC [10], and Illumina GAII platforms, for melanoma [10] and prostate cancer [11]. The lung cancer mutations [8] were determined using 31- to 35-base mate-paired reads from DNA nanoarrays produced from adsorbing sequence substrate to silicon substrates with grid-patterened arrays. To determine somatic mutations that are located in 3′UTRs, we compared the location of the mutation with the start and end locations of 3′UTRs of RefSeq genes from the UCSC genome browser [67], [68]. When necessary, we used the liftover tool in the Galaxy web-server [69] to convert genomic locations to the GRCh37/hg19 assembly of the human genome. To determine the frequency of each class of substitution, we selected only the somatic mutations that were single base substitutions from the list of somatic mutations in 3′UTRs as well as the complete list of somatic mutations across the entire genome from the supplementary information of the original papers for each of the cancers. To examine the relative location of somatic mutations within 3′UTRs, we first removed mutations that were located in multiple RefSeq genes that had different 3′UTRs.

### miRNA target sites altered by somatic mutation

We collected the sequences of the 3′UTR of all RefSeq genes using the UCSC Genome Browser. For each somatic mutation within a 3′UTR, we then created two sets of sequences, one containing the reference allele at the location of the somatic mutation and one containing the mutant allele. We then used two methods to identify somatic mutations that impacted putative miRNA target sites. First, we used TargetScan 6.0 [31] to calculate the impact of somatic mutations on the context+ score for the interaction between the 3′UTR sequence and all human miRNAs included in miRBase release 18 [37]. We also determined somatic mutations that impact binding to six miRNA seed classes [36], namely, 8mers (bases 1–8 of the miRNA), 7merA (bases 1–7), 7merB (bases 2–8), 6merA (bases 1–6), 6merB (bases 2–7), and 6merC (bases 3–8). We determined somatic mutations in 3′UTR sequences that disrupted, created, and modified potential target sites with perfect Watson-Crick complementarity to the miRNA seeds. Target sites found in the reference sequence and not the mutant sequence were disrupted by the somatic mutation, while target sites found in the mutant sequence and not the reference sequence were created by the somatic mutation. Target sites with different seed match types in the reference and mutant sequences (e.g., a reference sequence with a 6merA match to a miRNA that becomes a 7merA match in the mutated sequence) were modified by the somatic mutation (Table S1).

To help identify somatic mutations that altered functional mRNA-miRNA interactions, we collected miRNA expression data from several sources and added these data to Table S1. First, to identify miRNAs that are expressed in any tissue, we used the total number of RNA-Seq reads for mature miRNAs from all experiments included in miRBase release 18 [37]. Additionally, we collected tissue-specific mature miRNA expression from miRBase for melanoma and miRNA sequencing experiments by Landgraf et al. [70] for lung, SCLC, and prostate cancer. Tissue-specific miRNA expression in melanoma was determined by totaling the number of reads for each miRNA from 11 melanoma experiments included in miRBase. Tissue-specific miRNA expression for lung cancers (both lung and SCLC) and prostate cancer was determined by totaling the number of miRNA reads from 4 lung adenocarcinoma samples and 1 prostate sample, respectively.

### Linking somatic mutations with associations studies

To link somatic mutations that alter miRNA targeting with the results of association studies, we collected high ranking markers from association studies of cancer from dbGaP [71], the NHGRI GWAS Catalog [72], and the Cancer GAMAdb (http://www.hugenavigator.net/CancerGEMKB/caIntegratorStartPage.do). We first determined if the binding sites that were created or disrupted by these somatic mutations were also altered by germline mutations by identifying germline mutations from dbSNP build 132 [73], [74] that were located within seed matches in the mRNA sequences. We then calculated the distance between the target site containing the mutations and the association study markers and examined the linkage disequilibrium (LD) blocks of all markers that were within 100 Kb of an altered target site using Haploview [75]. For all but one highly ranked marker near a mutated target site, the association study was performed in a European population, and we obtained LD blocks using data from the CEU+TSI population from HapMap Project 2, release 27. The remaining GWAS marker (rs1247860) was associated with a cancer phenotype in a Han Chinese population [76]; we used the CHB population in Haploview and determined that no target sites containing somatic mutations were in LD with the marker. For germline mutations contained in the 1000 Genomes Project [47], we calculated the R^2^ or the correlation between the GWAS marker and the germline mutations within the LD block using SNAP [77].

## Supporting Information

Table S1 Impact of somatic mutations on miRNA target sites.
(XLS)
